# The metagenomics of soil bacteria and fungi and the release of mechanical dormancy in hard seeds

**DOI:** 10.3389/fpls.2023.1187614

**Published:** 2023-06-27

**Authors:** Yu Wu, Xiao-Rui Sun, Hugh W. Pritchard, Yong-Bao Shen, Xiao-Qin Wu, Chen-Yin Peng

**Affiliations:** ^1^ College of Forestry, Nanjing Forestry University, Nanjing, Jiangsu, China; ^2^ Co-innovation Center for Sustainable Forestry in Southern China, Southern Tree Inspection Center National Forestry Administration, Nanjing, Jiangsu, China; ^3^ Kunming Institute of Botany, Chinese Academy of Sciences, Kunming, Yunnan, China; ^4^ Royal Botanic Gardens, Kew, Wakehurst, Ardingly, Haywards Heath, West Sussex, United Kingdom

**Keywords:** hard coat seeds, mechanical dormancy breaking, soil microorganisms, seed viability, species identification, microbial scarification and cracking in pericarp

## Abstract

Persistence in the soil is a function of seed physiology, particularly non-germination and inherent lifespan. However, for seeds with mechanical dormancy, non-germination is also a function of the composition and activity of the soil microbiota. We attempted to screen out microorganisms in the soil that can specifically and rapidly decompose the hard fruit pericarps of *Tilia miqueliana* Maxim., a unique native tree species in China. Using the classical replica plating method, more than 100 different culturable microorganisms that could rapidly erode the pericarp were collected from the surface of pericarps under different culture conditions. At the same time, we successfully extended the concept of metagenomics and applied it to the identification of mixed artificial cultures. The decomposition process of the pericarps in soil was also simulated artificially. The physical and chemical data suggested a potential mechanism of microbial scarification and cracking in pericarp, whilst the embryos inside the eroded fruits retained good viability. Our discoveries could pave the way for the removal of physical and mechanical obstacles that prevent hard coat seeds from germinating. We anticipate that the use of this technology will improve the germination of other hard coat seeds. More research is needed to investigate the impacts on other seeds. The findings of this research can inform the design of experiments on the seed ecology of persistence.

## Introduction

Seed dormancy is a physiological phenomenon during seed development that impacts the timing of germination and seedling emergence, and contributes to persistence the survival of seeds in the environment once they have reached maturity (Long et al., 2015). Species with delayed and slow germination levels and rates can have advantages unpredictable ecosystems, like those fire- or flood-prone, among other) (Sales et al., 2013; Qasem, 2019; Fajinmi et al., 2021). However, in species recovery and seedling recruitment in agriculture and forestry, as well as landscape development or habitat restoration, slow germination and low germination rates due to seed dormancy are not a good thing. But in the context of restoration where rapid plant re-establishment is critical to prevent further degradation, dormancy can pose a significant challenge (Turner et al., 2013). Additionally, specialized dormancy and germination requirements can also constrain efforts to increase the scale and diversity of ex situ native seed production, limiting the ability of practitioners to work with multiple species at larger scales (Miller et al., 2017; Ladouceur et al., 2018). Many studies have focused on the means of removing seed dormancy, with the corresponding measures dependent on the type of seed dormancy present. Based on the dormancy classification of Baskin and Baskin (2004), which is reviewed comprehensively in Finch Savage and Leubner Metzger (2006), seed dormancy can be broadly divided into (i) physical (PY), in which seeds possess an impermeable coat that prevents water reaching the embryo; (ii) physiological (PD), in which involves the embryo or the surrounding endosperm tissues; (iii) morphological (MD), in which the embryo is not fully developed at the time of seed dispersal and requires time to grow; (iv) morphophysiological (MPD), in which the embryo is undeveloped and a hormone imbalance inhibits further development and germination; and (v) combinational (PY+PD), in which seeds possess a physical barrier to water uptake as well as physiological dormancy. Reviews of the mechanisms underlying the most commonly occurring dormancy types are available: physical dormancy (Baskin and Baskin, 2004) and physiological dormancy (Finch Savage and Leubner Metzger, 2006; Finkelstein et al., 2008). Among them, PD is the most common form of dormancy in species (Baskin and Baskin, 2004; Baskin and Baskin, 2021). The great diversity in kinds of seed dormancy: a revision of the Nikolaeva–Baskin classification system for primary seed dormancy (Penfield, 2017), involves the embryo or the surrounding endosperm tissues. PD operates at three levels: deep, intermediate and nondeep. Embryos excised from nondeep PD seeds produce normal seedlings (Finch Savage and Leubner Metzger, 2006). In addition, PD seeds can be delayed in germinating by a mechanical constraint (fruit or seed coat dormancy) imposed by the embryo-covering layers that must be overcome by the growth potential of the embryo (Manz et al., 2005). Mechanical dormancy is commonly imposed in ‘seeds’ with a hard mechanical barrier associated with the seed-covering layers (woody or stony fruit wall) (Hill, 1933). Note that this condition is different from PY seeds, MD seeds are often permeable to water However, imbibition alone does not ensure germination.The seed-covering layers has to be reduced or removed. It where predetermined breaking points facilitate tissue rips before germination, for example the pericarp (Finch Savage and Leubner Metzger, 2006).

For *Tilia miqueliana* ‘seed,’ the strength of the pericarp and carpel are the most important factors that hinder the cracking and germination of the seed (Paulsen et al., 2013). Cracking is needed for breaking mechanical dormancy, which is first step needed for germination. In the natural environment, in the soil, the process of weakening a hard pericarp can take more than one year (Baskin and Baskin, 2014) and is affected by many factors. The degree of pericarp decay before falling to the soil can vary amongst fruits. Also, the rate of pericarp decay differs with soil type (Leck, 2012) and microorganisms that can decompose litter such as leaves, wood and roots (Chomel et al., 2016). Soil, and seed-based, microorganisms are also likely to be involved in the cracking process of hard pericarps. It has been reported that fungi can release mechanical dormancy by biomechanically breaking the pericarp of *Lepidium didymum* (Sperber et al., 2017). However, little is known about the relationships between beneficial and pernicous microorganisms (fungi and bacteria) and seed quality (Liu et al., 2022).Some of microorganisms stimulated seed germination, seedling health and growth (Shade et al., 2017; Del Carmen Orozco-Mosqueda et al., 2020; Liu et al., 2022). For example, wild Panax plants adapt to their thermal environment by harboring abundant beneficial seed endophytic bacteria (Liu et al., 2022). Some of microorganisms caused seed deterioration, including for seeds retained the hard-seeded characteristic. Microbial microbial scarification is not necessarily a bad thing for hard coat seeds. Under natural conditions, the resistance to microbial attack may act in conjunction with seed hardening to maintain the longevity of *Abutilon theophrasti* seed in the soil (Kremer et al., 1984). So it would be interesting to be able to use this phenomenon to artificially control the extent of microbial scarification of hard coat seeds, to control the balance.

*Tilia miqueliana* Maxim., is a unique tree species native to east part of China (Tang et al., 2009). The species is classified as rare and endangered and is under national key protection. The fruits of *T. miqueliana* will not crack even after soaking in sterile deionized water for 2-3 months. Under natural conditions, the fruits are deeply dormant. It does not germinate until 2-3 years after falling into the soil, and the germination rate is low (Shi and Zhu, 2008). The dormancy in *T. miqueliana* fruits was combinational, with the mechanical dormancy caused by hard pericarp, and the physiological dormancy caused by endosperm. The pericarp of *T. miqueliana* is composed of highly lignified thick-walled cells and thin-walled cells. Microorganisms have a decomposition effect on pericarp cells. In this study, the mechanism of pericarp cracking was revealed by the action of microorganisms on the pericarp of *T. miqueliana*. This makes the species a good model system on which to study the microorganisms that contribute to loss of dormancy in the natural environment. After observation of soil sample plots of *Tilia miqueliana* we selected several sampling sites with rapid litter degradation, and then artificially simulated the decomposition process of pericarps from *T. miqueliana* seed in soil. The purpose was to understand the mechanism of pericarp cracking and seed germination in this species. We screened out microorganisms that can specifically and rapidly decompose the pericarp tissue. These target microorganisms were then artificially cultured and applied to *T. miqueliana* seed to speed up the artificial cultivation process. Thus, application of this method would have positive effects on the germination of other hard coat seeds: it will be a breakthrough in the removal of the mechanical restraint that is crucial to the germination of hard coat seeds.

## Materials and methods

### Soil sample collection

The soil was sampled at Nanjing Forestry University in Jiangsu Province, China, on September 15, 2020. The sampling sites of the three treatment groups were all located in the vegetation area on the east slope of the Biotechnology Building (**Table 1**). Soil samples were collected from rhizosphere soils of *Sophora japonica* (CHS1), *Lycoris radiata* (SSH2), and *Pinus* sp. (SSS3). Three separate soil samples (replicates) were collected from sampling sites in each treatment group and stored independently. The sampling method is to remove the topsoil within half a meter of the rhizosphere and excavate to a depth of 20-30 cm. Then, the rootlets and the adherent soils from roots were placed in sterile polyethylene bags and stored at 4°C. The physical and chemical properties of the soil were determined according to international standard methods (Nortcliff, 2002).

**Table 1:** Comparison of chemical and physical properties of soil samples from different collection points.

Sampling point of the soil		Latitude and longitude of the sampling point	Chemical and physical properties of soil									
			TC %	SOC g/kg	TN g/kg	AN mg/kg	TP g/kg	AP mg/kg	AK mg/kg	HM g/kg	pH	EC μs/cm
CHS1	*Sophora japonica*	32°4´43" N, 118°48´39" E	2.309	30.042	2.643	147.322	1.068	22.008	275.059	8.951	6.48	157.6
SSH2	*Lycoris radiata*	32°4´43" N, 118°48´38" E	2.786	41.367	2.658	139.259	0.814	23.864	217.167	9.420	6.66	148.6
SSS3	*Pinus Sp.*	32°4´43" N, 118°48´37" E	2.759	41.197	2.686	251.952	0.808	16.922	287.520	12.102	7.60	228.0

TC, total carbon; SOC, soil organic carbon; TN, total nitrogen; AN, available nitrogen = alkali-hydrolyzable nitrogen; TP, total phosphorus; AP, available phosphorus; AK, available potassium; HM, humus (Total carbon of humus); pH = Pondus Hydrogenii; EC, electrical conductivity. CHS1, SSH2, and SSS3 represent soil samples collected from rhizosphere soils of Sophora japonica, Lycoris radiata, and Pinus sp., respectively.

### Screening of microorganisms from soil that can erode *T. miqueliana* fruits

After the soil was sifted to remove large particles, stones, and other debris, soil samples were finely mixed with a grinder. All three soil samples were treated according to the following soil treatment scheme. Four soil sample treatments were used to screen out as many microorganisms as possible that could erode the outer pericarp: (1) no water was added to the original soil; (2) water was added to the original soil (moisturising); (3) water was added to a small amount of soil and then the mud was removed (only the turbid liquid was left); and (4) water was added to a small amount of soil and the mud was conserved (**Table 2**).The control group was fruits without adding soil and stored at room temperature. The experimental design took the form of A-B-C: sampling sites A:1–3 (representing three soil samples, respectively); sample treatments B:1–4 (representing four processing methods, respectively); and experiment repetition C:1–3 (representing three groups of identical replicates, respectively). Each replicate included 20 *T. miqueliana* fruits from a year with a full grain harvest. The above treatment groups were kept at 25 °C and 37 °C to screen for representative fungi and bacteria (Mallik, 2001; Liu et al., 2012). Changes in fruits in each soil treatment group were observed and recorded, and fruits with obvious signs of an eroded outer pericarp were finaMenglly screened out.

**Table 2:** Treatment methods for screening microorganisms from soil.

Name of groups		Number of groups	Treatment
1	Original soil with no water	A-1-1; A-1-2; A-1-3	Normal soil, normal moisture
2	Original soil with water	A-2-1; A-2-2; A-2-3	Moist soil, more moisture
3	Add water to a small amount of soil and then remove the mud	A-3-1; A-3-2; A-3-3	Muddy water, less nutrients, less moisture (Only the turbid liquid is left)
4	Add water to a small amount of soil and reserve the mud	A-4-1; A-4-2; A-4-3	Mud, rich in nutrients, more moisture

Sampling sites A: 1-3 (Represents three soil samples respectively); Sample treatments B:1-4 (Represents four processing methods respectively); Experiment repetition C:1-3 (Represents three groups of identical replicates respectively).

### Functional verification of microorganisms that can erode *T. miqueliana* fruits

According to the replica plating method (Lederberg and Lederberg, 1952), screened fruits were picked up with sterilised tweezers, and the eroded side of each seed was inoculated in contact with potato dextrose agar (PDA) and Luria-Bertani (LB) medium (Lobato et al., 2010; Zheng et al., 2019). After several rounds of separation and purification, all related microorganisms in each treatment group were isolated and their pure cultures were obtained. The experiment was then designed according to Koch’s postulates (King, 1952). The fruits of *T. miqueliana* were sterilised with 75% alcohol for 20 seconds, then washed with sterile deionized water twice, and the surface was disinfected. The sterilised healthy fruits of *T. miqueliana* were then inoculated into the pure cultures (culture dishes, six fruits per dish) of these microorganisms for secondary microbial scarification. Observe which microorganism could erode the fruits. These eroded fruits were selected, the microorganisms on them were isolated and purified in PDA and LB medium, and then these microorganisms were identified. Each treatment included six technical replicates (n = 6), each of which contained three biologicalreplicates in each treatment (Cooney, 1979).

### Characteristics of the pericarp structure of *T. miqueliana* fruits treated with target microorganisms

To explore the effects of target microorganisms on pericarp structure, we recorded microbial scarification and cracking in pericarps of *T. miqueliana* fruits under the influence of the target microorganisms. We also measured the viability of seeds (Copeland and McDonald, 1999). The seed viability was determined with TTC (2,3,5-Triphenyte-trazolium chloride) staining approach proposed by International Seed Testing Association (ISTA) (2018). The dehydrogenase in the viable embryo could reduce TTC to insoluble red TTF. If the embryo dies, or the embryo exhibits reduced viability, it cannot be stained or stained shallowly. Thus, by the location or the depth of the staining, the viability of the seed can be determined. Because water cannot penetrate through the seed coat, and the seed coat is difficult to remove, all intact seeds were longitudinal-cut treated, then placed in the 50ml beaker. 0.5% TTC solution was added and stained in the dark incubator at 35 °C for 6h. After the dyeing process, the longitudinal-cut seeds were cleaned with water 2-3 times for subsequent observation. All or the majority of the seed embryos dyed red refer to viable seeds, and those not stained refer to dead seeds. The seed viability rate was computed by equations proposed by ISTA as follows:


$$\text{Seed viablity rate \%}=\frac{\text{Viable embryos}}{\text{Total number of embryos}}\times 100$$

where seed viablity rate = percentage of viable embryos with all or most dyed red in the overall number of embryos. The final seed vigor rates were the means of 4 replicates ± standard deviation (SD).

To explore the mechanism by which microorganisms cause microbial scarification and cracking in pericarp, SEM (FEI Quanta 200, Hillsboro, OR, USA) images were collected to observe the changes in cell characterisation structure on the outer and inner surfaces, and along a cross section of the pericarp (Shehata and Loutfy, 2006). A 2-mm^2^ piece of the pericarp was mounted with double-sided tape on the sample stage of a scanning electron microscopy (SEM) (Thermo Fisher Scientific, Waltham, MA, USA). Samples were then gold-coated using a gold sputter coater (HITACHI E-1010, Tokyo, Japan) and observed by SEM in the high vacuum mode. Images were captured at 15 kV.

At the same time, the Vickers hardness index was assessed to determine the hardness of the pericarp (Grochowicz et al., 1994). The hardness of the pericarp was measured using a Vickers hardness machine (Falcon 507, Innovatest, Maastricht, The Netherlands). Eight seeds from the control and liquid N (40 s) groups were used in these analyses. After the outer surface of the pericarp was polished, the fruit was fixed with a clamp. A rhombus-shaped indentation was pressed into the pericarp’s outer surface using a diamond square pyramid with a vertex angle of 136°. A loading force of 10 gf was used and maintained for 10 s. The pressure was calculated based on the pressure per unit surface area of the indentation. The length of the two diagonal lines of the indentation was measured using the microscope on the instrument, and the software automatically displayed the hardness value. The anthrone, a tricyclic hydrocarbon (C14H10O), is generally used for cellulose assay (Dubois et al., 1956). This method consisted of adding anthrone solution (0.05 to 0.20%) in concentrated sulfuric acid to an aqueous solution of pericarp fibers (previously digested by sulfuric acid). The absorbance of the green color of the solution is measured using a UV–Vis spectrophotometer LAMDA650 (PerkinElmer, USA) at 620 nm and it is proportional to the cellulose content of the sample.

Ligin was determined by the modified acetyl bromide procedure of liyama and Walllis (Iiyama and Wallis, 1988) except that 10-15 mg of pericarp was weighed into 4 mL brown vials and 2.0 mL of acetyl bromide in glacial acetic acid (1:3, v/v) containing perchloric acid (70%, 0.08 mL) was added. After digestion, the samples were transferred, with the acid of acetic acid, to 50 mL volumetric flasks containing 2 _M_ sodium hydroxide (5 mL) and acetic acid (12 mL).

The 3, 5-dinitrosalicylic acid (DNS) was used to hemicellulose assay (Deshavath et al., 2020). Take 0.10g of pericarp and place it in a beaker (100 mL), add calcium nitrate solution (10 mL, 80%), boil for 5 min, then dilute and filter, and wash the precipitate three times with distilled water. After drying at 80 °C, transfer to a tube (15 mL), add HCL (10 mL, 2mol/L), boil for 45 min, transfer all to a triangular flask (150 mL), add one drop of phenolphthalein, neutralize with NaOH until it turns exactly rose colored, and filter. Wash the filter residue with distilled water and retain the filtrate. Add 1.5 mL of DNS (3,5-dinitrosalicylic acid) solution to 2 mL of filtrate, boil for 5 min, cool and bring to volume to 15 mL, and measure absorbance at a wavelength of 540 nm.

### Metagenomic analysis of soil microbial diversity and target microorganisms

First, to explore the microbial diversity in the three soil samples, we measured the differences between the fungi and bacteria in the three soil samples (The natural soil microbiome) using the soil metagenomic sequencing method (Ko et al., 2012). After preliminary tests (After the section, Functional verification of microorganisms), several fungi and bacteria in soil samples kept at 25 °C and 37 °C that could erode or cause the pericarp to crack were screened out. We artificially simulated the presence of microorganisms in soil, collected pure cultures of these microorganisms, and then mixed them, treating the mixture as an artificial simulation of the soil microbial community (The artificially mixed soil microbiome). Then soil microbial metagenomic sequencing method was used again to quickly confirm the taxonomic status of the desired target microbes isolated and purified from the soil. At the same time, it can also verify whether these isolated target microbes are from the three soil samples. Here we refer to this approach as metagenomics identification.

### Screening of identification of target microorganisms

Morphological and molecular methods were used to identify the representative purified microorganisms that could erode *T. miqueliana* fruits (Cooney, 1979; Nagashima et al., 1998). We recorded the micro-morphological characteristics of target fungi and bacteria and examined the cultured strains under a compound microscope (Axio Imager M2.0; Zeiss, Jena, Germany) equipped with a digital camera (AxioCam HRc, Zeiss); SEM images were also used. The colonies were dehydrated in graded ethanol solutions and subjected to critical point drying with liquid CO_2_ (K850, EmiTech, Lewes, UK). The fungi and bacteria were then examined using SEM after sputtering with gold (E-1010, Hitachi, Tokyo, Japan) (Taylor-George et al., 1983).

DNA was extracted and purified according to the method set out by Cubero et al. (Cubero et al., 1999). The 16S rDNA region was amplified and sequenced with the primers 27F and 1492R and the ITS sequences (internal transcribed spacer) were confirmed with the primers ITS1 and ITS4. The polymerase chain reaction (PCR) amplification consisted of 3 min at 94°C, followed by 35 cycles of 30 s at 94°C, 30 s at 58°C and 1 min at 72°C, with a final 10 min at 72°C. The PCR amplicons were sequenced at Beijing Huada Gene, China. All of the new sequences used for our analyses were deposited in GenBank.

In the phylogenetic analyses, the sequences that best matched the 16S rDNA and ITS regions of six target microorganisms were selected after a blast search of the National Center for Biotechnology Information (NCBI) database (http://www.ncbi.nlm.nih.gov). These sequences were aligned using BioEdit software. The aligned datasets were analysed using the ML method, and ML phylogenetic trees with 1000 bootstrap replications were constructed separately for 16Sr DNA and ITS using MEGA (6.0) (El-Esawi et al., 2018). Bayesian Inference phylogenies were inferred using PhyloSuite v1.2.1 (Ko et al., 2012; Zhang et al., 2020).

### Statistical analyses

Microsoft Excel 2003 (Microsoft, Redmond, CA) and SPSS (version 18.0, IBM, New York, NY) were used to collate and analyse the experimental data. Data are expressed as a mean ± standard deviation (SD). One-way analysis of variance (ANOVA) and Duncan’s multiple range test (two-tailed) were used to perform significant differences and multiple comparison analyses (no adjustment). Statistical differences were significant at the 0.05 level.

### Code availability

The natural soil microbiome. BioProject: PRJNA948580 (soil microbial diversity). For bacteria: Soil 1 CHSNa1-1-16S (TaxID: 410658); BioSample: SAMN33907250; SRA: SRR23969042. Soil 2 SSHNa2-2-16S (TaxID: 410658); BioSample: SAMN33907251; SRA: SRR23969041. Soil 3 SSSNa3-3-16S (TaxID: 410658); BioSample: SAMN33907252; SRA: SRR23969040. For fungi: Soil 1 CHSNa1-1-ITS (TaxID: 410658); BioSample: SAMN33907253; SRA: SRR23969039. Soil 2 SSHNa2-2-ITS (TaxID: 410658); BioSample: SAMN33907254; SRA: SRR23969038. Soil 3 SSSNa3-3-ITS (TaxID: 410658); BioSample: SAMN33907255; SRA: SRR23969037.

The artificially mixed soil microbiome. BioProject: PRJNA951536 (soil microbial diversity). For bacteria: 25 °C 25Ar1-1-16S (TaxID: 410658); BioSample: SAMN34045120; SRA: SRR24082341. 37 °C 37Ar2-2-16S (TaxID: 410658); BioSample: SAMN34045121; SRA: SRR24082340. For fungi: 25 °C 25Ar1-1-ITS (TaxID: 410658); BioSample: SAMN34045122; SRA: SRR24082339. 37 °C 37AR2-2-ITS (TaxID: 410658); BioSample: SAMN34045123; SRA: SRR24082338.

Sequences of ITS gene region from isolates fungi F1, F2, F3 and Sequences of 16Sr RNA gene region from bacteria B1, B2, B3 have been sequenced and deposited in Gen Bank (https://submit.ncbi.nlm.nih.gov/subs/genbank). Accession number of ITS from 3 isolates fungi: F1 = OP117393, F2 =OP117394, F3 = OP117395; Accession number of 16S rRNA from 3 isolates bacteria: B1 = OP117441, B2 = OP117442, B3 = OP117443.

## Results

### Comparison of the chemical and physical properties of soil samples

The soil sampling points and soil chemical and physical index data were listed in **Table 1**. Differences were observed among the three soil samples in terms of soil organic carbon (SOC), available nitrogen (AN), available phosphorous (AP), available potassium (AK), electrical conductivity (EC), humus (HM, Total carbon of humus), Pondus Hydrogenii (pH), and other indexes. The soil in the three sample plots was very different, with each soil having its own representative characteristics.

### Screening of fruits with microbial scarification and cracks

In this experiment, the decomposition process of the pericarps from *T*. *miqueliana* seed in soil was simulated artificially. Each soil sample treatment group included 20 *T. miqueliana* fruits from a year with a full grain harvest.

**Figure 1:**
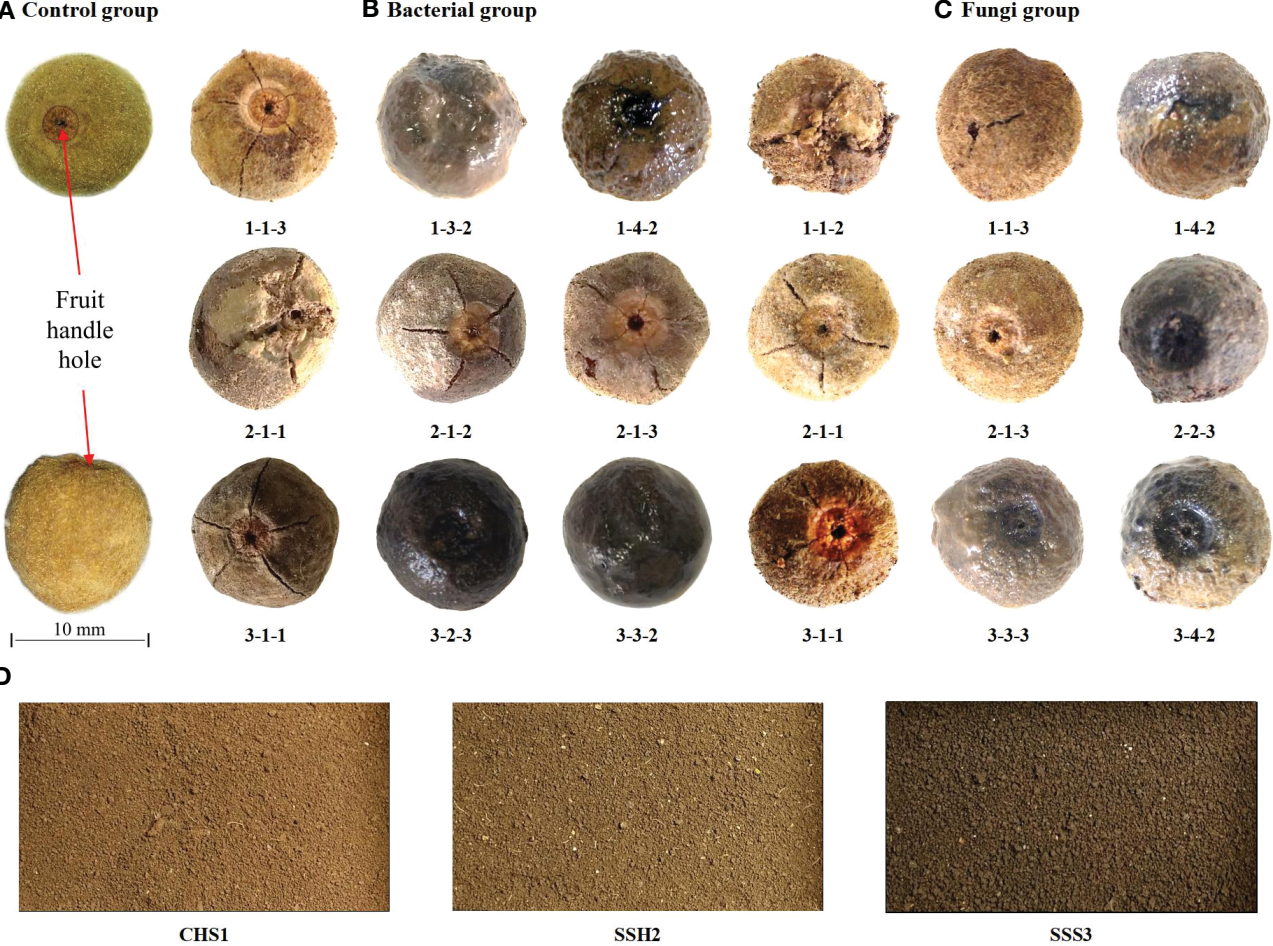
The fruits of *Tilia miqueliana* with erosion and cracks screened from different treatment groups. Control group **(A)**, bacterial group **(B)** and fungi group **(C)**. The bar of A, B, and C was the same size with 10 mm.CHS1, SSH2, and SSS3 represent soil samples collected from rhizosphere soils of *Sophora japonica, Lycoris radiata*, and *Pinus* sp., respectively. The figure shows morphology and color of the soils at three sampling sites **(D)**.

After 39 days of treatment in the different treatment groups, representative treatment groups were selected, and the fruits were photographed and described. Pericarps eroded and cracked in both the 25 °C and 37 °C treatment groups. The control group was fruits stored at room temperature (**Figure 1A**). At 25 °C, fruits with eroded pericarps were observed in the 1-4-2, 2-2-3, 3-3-3 and 3-4-2 treatment groups, while fruits with cracked pericarps were observed in the 1-1-2, 1-1-3, 2-1-1, 2-1-3 and 3-1-1 treatment groups (**Figure 1B**). At 37 °C, fruits with eroded pericarps were observed in the 1-3-2, 1-4-2 and 3-3-2 treatment groups, while fruits with cracked pericarps were observed in the 1-1-3, 2-1-2, 2-1-3, 2-2-1, 3-1-1 and 3-2-3 treatment groups (**Figure 1C**). The color and state of the soil samples used are shown in **Figure 1D**. Number 1, 2 and 3 are sampling point of the soil (**Table 2**).

### Soil microbial diversity in the three soil samples

**Figure 2:**
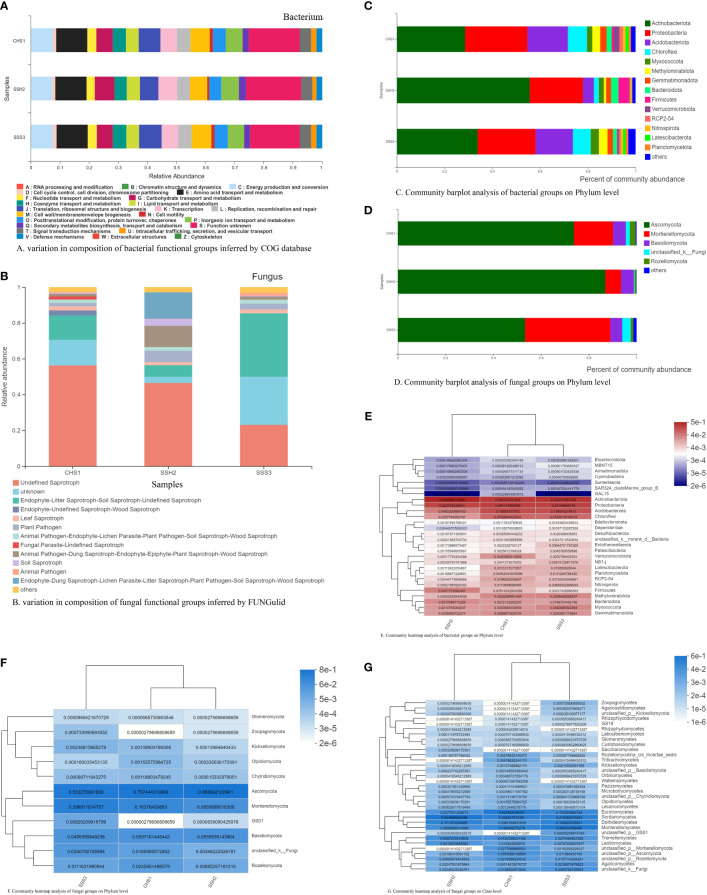
Functional groups composition **(A, B)** and community analysis of bacteria **(C, E)** and fungi **(D, F, G)** in three soils.

The Clusters of Orthologous Groups (COG) is a database for the homologous classification of gene products. The types and proportions of the various biological functions performed by bacterial flora in the three soil samples were relatively similar (**Figure 2A**). Together, these findings suggest that the bacterial flora may have had a high level of redundancy in their biological functions. FUNGuild is a tool for the functional classification and prediction analysis of fungal communities through the identification of micro-ecological guilds. The trophic mode of microorganisms includes pathotrophs, symbiotrophs and saprotrophs. Large numbers of saprophytic microbes were observed in all three soil samples (**Figure 2B**), including many potential target strains that could decompose plant fibres.

Because more bacterial species were observed than fungi species, to comprehensively investigate the presence of bacterial flora in each soil, the top four optimal bacterial flora (one more than fungi) in each soil were selected for comparative analysis. At the phylum level, the dominant flora in the three soil samples included Actinobacteriota, Proteobacteria, Acidobacteriota, Chloroflexi, Myxococcota, Methylomirabilota, Gemmatimonadota, Bacteroidota, Firmicutes, Verrucomicrobiota, RCP2-54, Nitrospirota, Latescibacterota, Planctomycetota and others (**Figure 2C**). However, the dominant bacterial communities differed slightly between the different soil samples (in types and proportions). In order of bacterial abundance, the top four optimal bacterial communities in each soil were: Soil No. 1, Actinobacteriota, Proteobacteria, Acidobacteriota and Chloroflexi; Soil No. 2, Actinobacteriota, Proteobacteria, Acidobacteriota and Firmicutes; and Soil No. 3, Actinobacteriota, Proteobacteria, Acidobacteriota and Chloroflexi. The top three optimal fungal flora in each soil were selected for comparison and analysis. At the phylum level, the dominant flora in the three soil samples included Ascomycota, Mortierellomycota, Basidiomycota, Rozellomycota, unclassified_k:Fungi and others (**Figure 2D**). However, the dominant flora differed slightly between the different soil samples. In order of fungi abundance, the top three optimal flora in each soil were: Soil No. 1, Ascomycota, Mortierellomycota and Basidiomycota; Soil No. 2, Ascomycota, Mortierellomycota and Basidiomycota; and Soil No. 3, Ascomycota, Mortierellomycota and Basidiomycota.

The results of the heat map of the bacterial community at the phylum classification level in the three soil samples revealed that the optimal dominant bacterial community types were relatively similar and included the four bacterial communities of Actinobacteriota, Proteobacteria, Acidobacteriota and Chloroflexi. Actinobacteriota and Proteobacteria were the common dominant bacteria in the three different soil types (**Figure 2E**). At the same time, the species grouping revealed that the four bacterial communities were relatively closely related to each other. The finding that they were clustered together may indicate that these dominant bacterial communities had the same survival strategy in the three soils. The sample grouping relationship between Soils No. 1 and No. 3 was relatively close, and the four bacteria groups with the highest abundance in the soil were Actinobacteriota, Proteobacteria, Acidobacteriota and Chloroflexi, which indicates that the similarity between the two soils was relatively close. In all three soils, the second dominant flora were also closely related and clustered together, suggesting that these dominant floras may have survived in the soil by another similar survival strategy. These bacterial communities include Firmicutes, Methylomirabilota, Bacteroidota, Myxococcota and Gemmatimonadota (**Figure 2E**). Ascomycota was the most dominant fungal flora among the three soil samples (**Figure 2F**). We used the class classification level (**Figure 2G**) in heat map of the fungal community, which produced clearer and richer data than the phylum classification level (**Figure 2F**), to further detail and analyse the most dominant fungal flora in the three soil samples. The optimal dominant community types in the three soils were relatively similar: Eurotiomycetes, Sordariomycetes, Dothideomycetes and Mortierellomycetes. Sordariomycetes was the dominant microbe in the three different soil types. The species grouping revealed that the four fungal communities were relatively closely related to each other. The fact that they were clustered together may indicate that these dominant communities had the same survival strategy in the three soils. The sample grouping relationship between Soils No. 1 and No. 3 was relatively close, and the three communities with the highest abundance in soil were Eurotiomycetes, Sordariomycetes and Mortierellomycetes, indicating strong similarity between the two soils.

**Figure 3:**
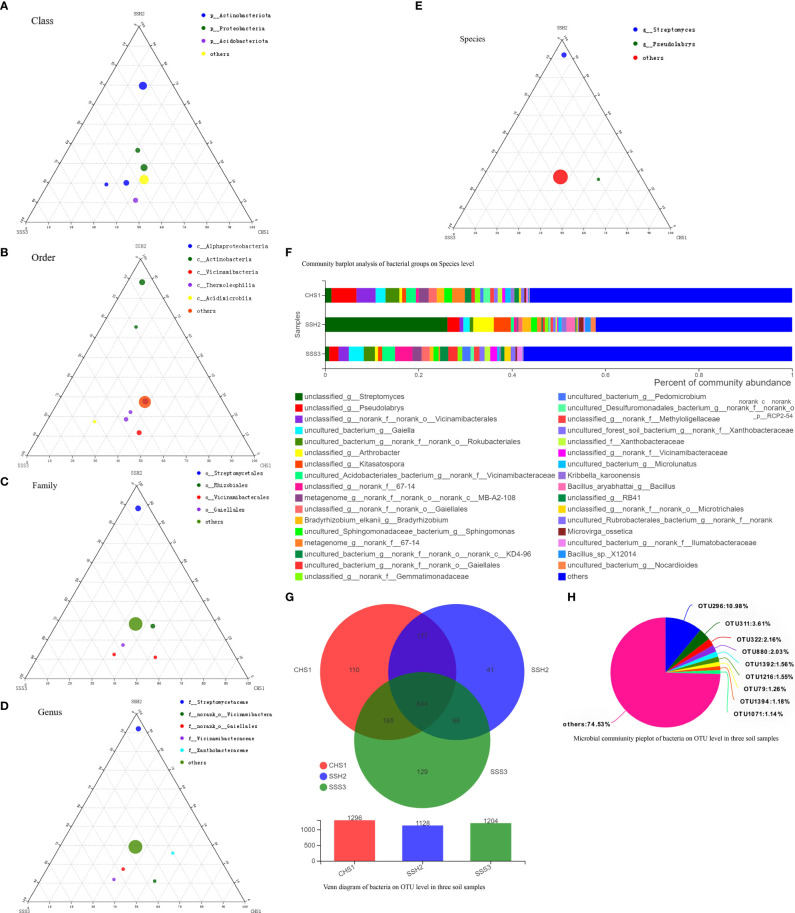
The Ternary Ternary phase map visualized the composition and distribution proportion of dominant bacterial species in the three soil samples **(A-E)**. Venn diagram of bacteria on OTU level in three soil samples **(F)**. Microbial commiunity pieplot of bacteria on OTU level in three soil samples **(G)**. Community barplot analysis of bacterial groups on Species level **(H)**.

**Figure 4:**
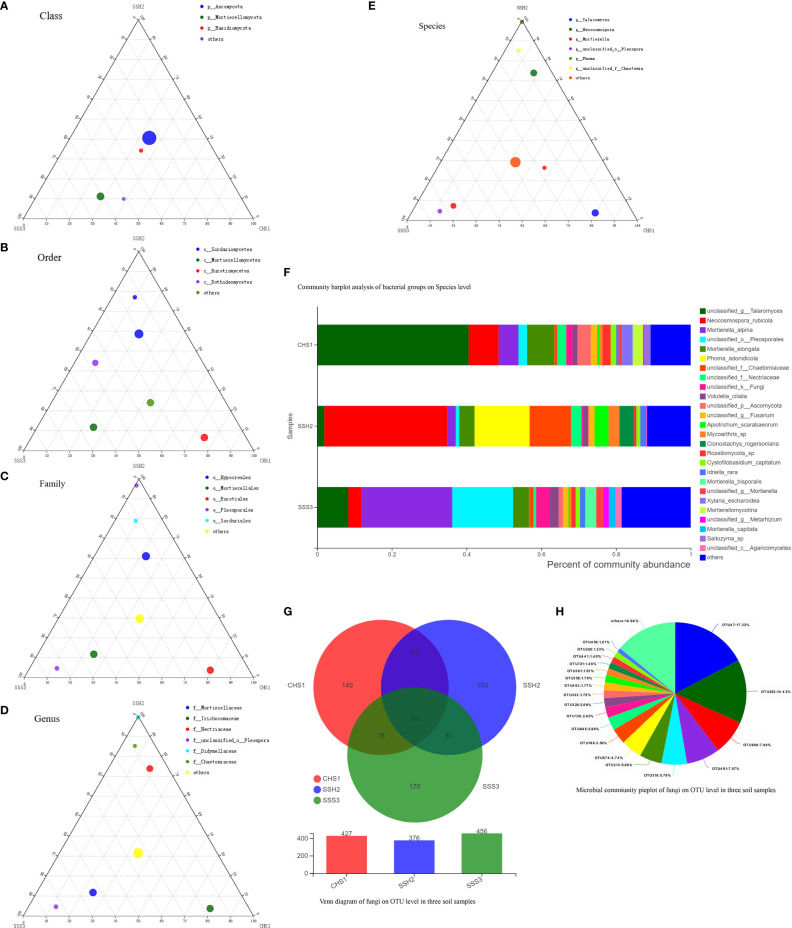
The Ternary Ternary phase map visualized the composition and distribution proportion of dominant fungal species in the three soil samples **(A-E)**. Venn diagram of fungi on OTU level in three soil samples **(F)**. Microbial commiunity pieplot of fungi on OTU level in three soil samples **(G)**. Community barplot analysis of fungal groups on Species level **(H)**.

A ternary phase diagram was constructed to explain the composition and distribution of the dominant bacterial (**Figures 3A-E**) and fungal (**Figures 4A-E**) species in the three soil samples. Soil No. 2 had a unique group of bacteria: class Alphaproteobacteria, order Streptomycetales, family Streptomycetaceae, genus *Streptomyces* and species unclassified-*Streptomyces*. This was different from SoilsNo. 1 and No. 2, which phylogenetically indicated the presence of the same branch at different taxonomic levels. The dot symbol close to the top of the triangle is the position where the Soil No. 2 is located (**Figures 3A–E**, **4A–E**). Each soil sample had a different dominant fungal flora, among which there was also a unique group of bacteria, for example order Eurotiales, family Trichocomaceae, genus *Talaromyces* and species unclassified–*Talaromyces* in Soil No. 1. Soil No. 2 had a unique group of bacteria: order Pleosporales, family Didymellaceae, genus *Phuma* and species *Phuma Adonidicola*. Soil No. 3 had a unique group of bacteria: order Pleosporales, family f:unclassified_o:Pleosporales, genus g:unclassified_o:Pleosporales and species s:unclassified_o:Pleosporales. Phylogenetically, the presence of different taxonomic levels of flora in each soil all pointed to the same branch.

At the genus and species classification level, the distribution of the dominant bacteria in the three soil samples was similar to that of the family level (**Figure 3F**). Among the three soils, except Soil No. 2 in which Streptomycetaceae and *Streptomyces* had a high abundance, the bacterial flora in the other soils had a wide variety and high abundance. The dominant flora differed slightly between the different soil samples, and the top three optimal flora in each soil were counted according to the order of bacterial abundance. *Pseudolabrys* was the dominant bacterial community in Soil No. 1, while it was difficult to define the taxonomic status of the second and third bacterial communities. *Streptomyces*, *Arthrobacter* and *Kitasatospora* were the dominant bacterial communities in Soil No. 2, while *Gaiella* was ranked second in Soil No. 3 and the classification status of the first and third bacteria was difficult to define. These results suggest that the different dominant bacterial communities have their own unique survival strategies and can survive in the same soil. The accuracy of fungal flora classification at the genus and species level was high (**Figure 4F**). At the species level, the dominant fungal flora in the three soil samples included *Apiotrichum*_*scarabaeorum*, *Clonostachys*_*rogersoniana*, *Cystofilobasidium*_*capitatum*, *Idriella*_*rara*, *Mortierella*_*bisporalis*, *Mortierella*_*capitata*, *Mortierella*_*alpin, Mortierella*_*elongata*, *Neocosmospora*_*rubicola*, *Phoma*_*adonidicola*, *Volutella*_*ciliata*, *Xylaria*_*escharoidea*, Mortierellomycotina, *Mycoarthris*_sp, *Saitozyma*_sp, *Rozellomycota*_sp, unclassified_p:Ascomycota, unclassified_c:Agaricomycetes, unclassified_o:Pleosporales, unclassified_f:Chaetomiaceae, unclassified_f:Nectriaceae, unclassified_g:*Talaromyces*, unclassified_g:*Fusarium*, unclassified_g:*Mortierella*, unclassified_g:*Metarhizium*, unclassified_k:Fungi and others. However, the dominant flora differed slightly between the different soil samples. In order of bacterial abundance, the top three optimal flora in each soil were: Soil No. 1, unclassified_g:*Talaromyces*, *Neocosmospora*_*rubicola* and *Mortierella*_*elongata*; Soil No. 2, *Neocosmospora*_*rubicola*, *Phoma*_*adonidicola* and unclassified_f:Chaetomiaceae; and Soil No. 3, *Mortierella*_*alpina*, unclassified_o:Pleosporales and unclassified_g:*Talaromyces*. When comparing these results with the soil microbial diversity results for bacteria, we speculated that the compatibility between the bacteria may be higher than that of the fungi.

Due to the low accuracy of bacterial flora at the classification level of genus and species, we constructed a Wynn diagram of the bacterial flora distribution at the operational taxonomic unit (OTU) classification level in the three soil samples to investigate the bacterial flora in the three soil samples (**Figure 3G**). Nine OTUs with a high common abundance in the three soil samples were found, and the proportion of these OTUs to the total OTUs ranged from 1.14% (OTU1071) to 10.98% (OTU296) (**Figure 3H**). As the smallest unit of classification, the OTU results should be similar to that of taxon species richness. The types and abundance of the optimal flora shown in **Figures 3E, F** were basically the same, which also complemented and verified the correctness of the results at the species level of classification. Most of these bacteria have a saprophytic function and are one of the main decomposer groups in nature. The accuracy of bacterial species identification was lower than that of fungi. Compared with the distribution of fungi at the same level in the soil, more floras were inaccurately classified at the genus and species classification levels. We also constructed a Wynn diagram of the distribution of fungal flora at the OTU classification level for the three soil samples (**Figure 4G**). Among the three soil samples, 19 fungal OTUs had a high common abundance. These OTUs accounted for between 1.01% (OTU456) and 17.33% (OTU47) of the total OTUs, with OTUs below 1% accounting for 14.04% (**Figure 4H**). These results may also indicate that fungi are more competitive than bacteria within families, genera and species.

### Selection of microorganisms capable of eroding or cracking pericarps

The results of a functional verification test revealed that the pure cultures of these screened microorganisms could erode the fruits of *T. miqueliana* again. More than 100 different microorganisms (50 fungi + 50 bacteria) with the ability to rapidly erode pericarps were initially screened. Second metagenomic sequencing results showed that 18 bacterial species were detected at 25 °C, four bacterial species were detected at 37 °C, and four genera (*Bacillus*, *Knoellia*, *Lysinibacillus* and *Streptomyces*) were detected as common bacteria in the two different temperature treatment groups, including seven species (**Table 3**). In addition, 39 fungal species were detected at 25 °C. There were five fungal species detected at 37 °C. Four genera (*Alternaria*, *Aspergillus*, *Penicillium* and *Talaromyces*) were detected as common fungi in the two different temperature treatment groups, including nine species (**Table 4**).

**Table 3:** Bacterial species detected by second metagenomic sequencing methods.

The treatment group being examined	The detected bacterial species
25 °C treatment group	*Brevibacillus_fluminis*, *Burkholderia_cenocepacia, Clostridium_beijerinckii*, *Methylobacterium_brachiatum*, *Mycobacterium_obuense*, *Paenibacillus_alginolyticus*, *Paenibacillus_xylanexedens*, *Paraburkholderia_terrae*, *Pseudomonas_nitroreducens*, *Sphingomonas_yunnanensis*, s:unclassified_g:_*Achromobacter*, s:_unclassified_g:_*Allorhizobium-Neorhizobium-Pararhizobium-Rhizobium*, s:_unclassified_g:_*Azospirillum*, s:_unclassified_g:_*Enterobacter*, s:_unclassified_f:_Enterobacteriaceae, s:_unclassified_g:_*Kosakonia*, s:_unclassified_g:_*Paenibacillus*, s:_uncultured_bacterium_g:_*Rhodopseudomonas*
37 °C treatment group	*Bacillus*_sp._Y1, *Microbacterium_esteraromaticum*, *Paenibacillus_barengoltzii*, *Pseudomonas_mosselii.*
Both 25 °C and 37 °C	*Bacillus_anthracis*, *Bacillus_velezensis*, *Streptomyces_lanatus*, s:_unclassified_o:_Bacillales, s:_unclassified_g:_*Bacillus*, s:_unclassified_g:_*Lysinibacillus*, s:_uncultured_bacterium_g:_*Knoellia*.

**Table 4:** Fungal species detected by second metagenomic sequencing methods.

The treatment group being examined	The identified fungal species
25 °C treatment group	*Albifimbria_verrucaria*, *Albonectria_rigidiuscula*, *Aschersonia_*sp., *Aspergillus_aculeatus*, *Beauveria_pseudobassiana*, *Bjerkandera_fumosa*, *Chaetomium_globosum*, *Cladosporium_anthropophilum*, *Cladosporium_delicatulum*, *Clonostachys_rogersoniana*, *Clonostachys_*sp., s:*Cutaneotrichosporon_curvatus*, s:_*Exophiala_pisciphila*, s:_*Fusarium_concentricum*, s:_*Hansfordia_pulvinata*, s*:_Latorua_caligans*, s:_*Lecanicillium_aphanocladii*, s:_*Malassezia_*sp., s:_*Neocosmospora_rubicola*, s:_*Penicillium_cordubense*, s:_*Penicillium_ovatum*, s:_*Penicillium_oxalicum*, s:_*Penicillium_simplicissimum*, s:_*Phanerochaete_chrysosporium*, s:_*Purpureocillium_lilacinum*, s:_*Rhizopus_arrhizus*, s:_*Sistotremastrum_guttuliferum*, s:_*Talaromyces_assiutensis*, s:_*Trichoderma_fomitopsis*, s:_*Trichoderma_neosinense*, s:_*Trichothecium_roseum*, s:_unclassified_g*:_Botryosphaeria*, s:_unclassified_g*:_Cladosporium*, g:_unclassified_f:_Metschnikowiaceae, s:_unclassified_g*:_Pestalotiopsis*, s:_unclassified_o*:_*Pleosporale*s*, s:_unclassified_g*:_Talaromyces*, s:_unclassified_g*:_Trichoderma*.
37 °C treatment group	*Aspergillus_minisclerotigenes*, *Hormographiella_aspergillata*, Lichtheimiaceae_sp., s:unclassified_f*:*Chaetomiaceae, s:unclassified_g*:Scedosporium*.
Both 25 °C and 37 °C	s:unclassified_g:*Alternaria*, s:unclassified_g:*Aspergillus*, *Aspergillus_terreus*, *Aspergillus_tubingensis*, *Penicillium_citrinum*, *Penicillium_sumatraense*, *Talaromyces_islandicus*, *Talaromyces_pinophilus*, *Talaromyces_wortmannii*

The taxonomic status of some microorganisms was unclear due to the limitations of the 16S and internal transcribed spacer (ITS) sequences in enabling accurate species identification. A total of 29 OTU taxa were detected in the bacteria group, 7 common OTUs were detected in the two different temperature treatment groups, 25 (18 + 7) OTUs were detected at 25 °C, and 11 (4 + 7) OTUs were detected at 37 °C. In addition, A total of 57 OTU taxa were detected in fungi group, 9 common OTUs were detected in the two different temperature treatment groups, 52 (43 + 9) OTUs were detected at 25 °C, and 14 (5 + 9) OTUs were detected at 37 °C. Almost all of the species detected in the second metagenomic test were found in the first metagenomic test.

### Changes in fruits treated with target microorganisms

**Figure 5:**
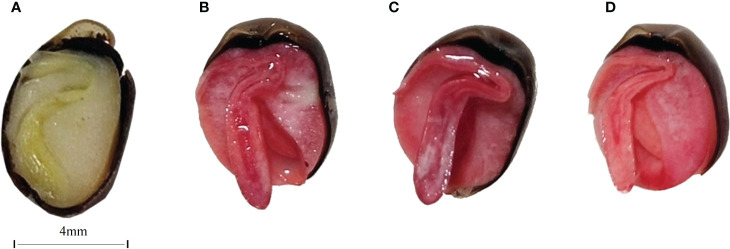
Longitudinal cross-section images of unstained CK **(A)**, CK**(B)**, 25 °C **(C)**, and 37 °C **(D)** treated group. The bar of A, B, C, and D was the same size with 4mm.

To investigate the survival rate of seed treated by target microorganisms, the viability of the seed was measured firstly. Seed viability was determined by 2,3,5-triphenyltetrazolium chloride (TTC) staining: an active seed was stained red, while an inactive seed was not stained. The results revealed that seed in the 25°C and 37°C treatment groups had a high viability (**Figure 5**). The radicles of the seed were almost stained red.

**Figure 6:**
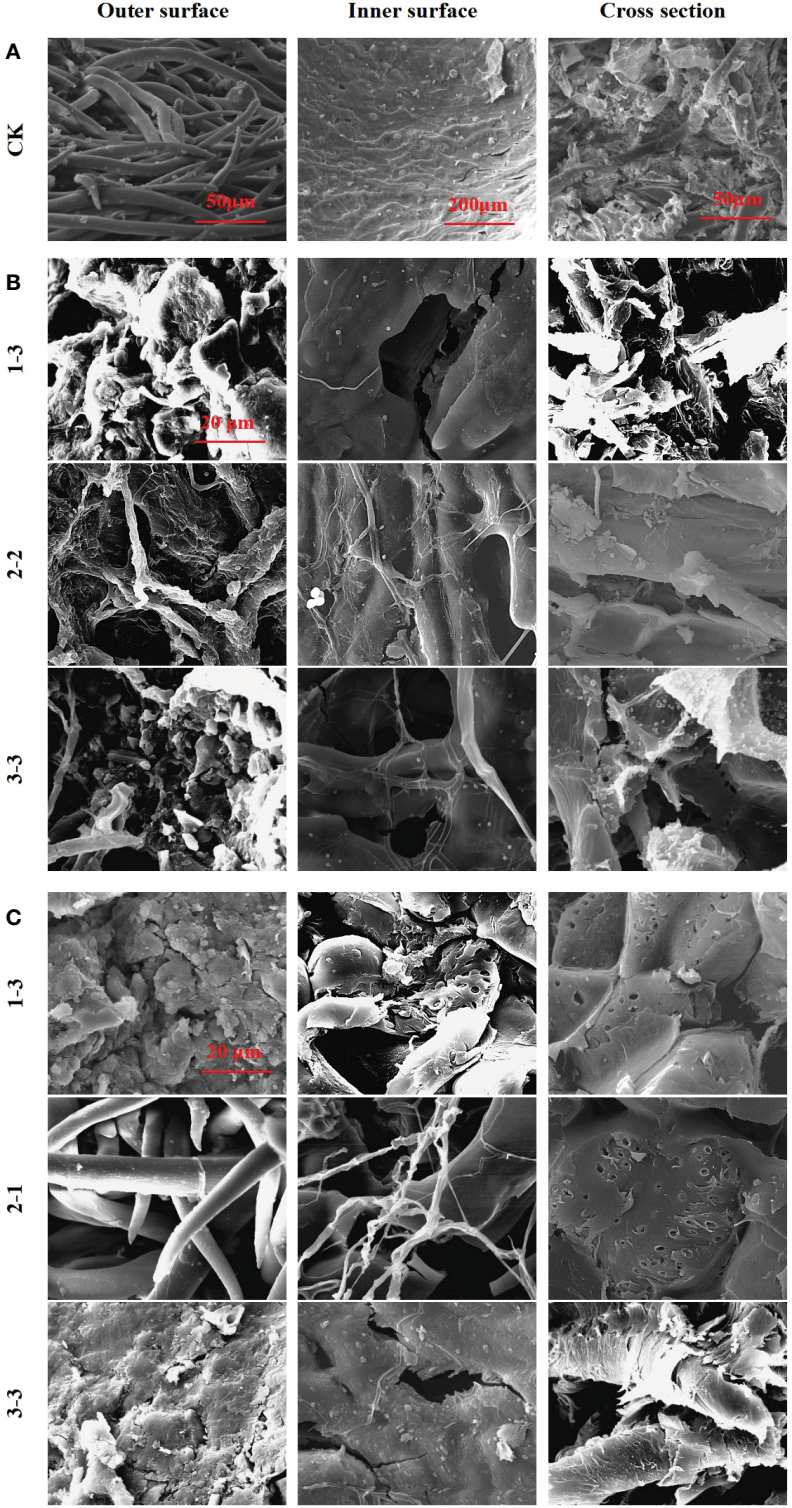
SEM images of pericarp form *Tilia miqueliana*. Control group **(A)**, 25°C treatment group **(B)** and 37°C treatment group **(C)**. The bar size of A (From left to right) was 50 μm, 200 μm and 50 μm. The bar of B and C was the same size with 20 μm.

To explore the mechanism by which microorganisms cause microbial scarification and cracking in pericarp, fruits from the 25°C and 37°C treatment groups were selected and them were viewed by using scanning electron microscopy (SEM) (**Figure 6**). Pericarp eroded and cracked in both the 25°C and 37°C treatment groups. At 25°C, fruits with eroded or cracked pericarps from the 1-3, 2-2 and 3-3 treatment groups were screened. At 37°C, fruits with eroded or cracked pericarps from the 1-3, 2-1 and 3-3 treatment groups were screened.

In the control group, intact fibrous villi could be seen on the outer surface of the pericarps without soil treatment. The inner surface presented a textured protrusion, and the inner wall structure was dense. The cross section revealed a coarse surface with small spikes, indicating that the pericarp fibre was not easy to cut (**Figure 6A**). The outer surface structure of the pericarps treated at 25°C was destroyed, and fibrous villi were wound by a mycelium, leading to serious damage. The inner wall structure of the inner surface was destroyed and holes had formed. The textured protrusions became blurred due to microbial scarification, with bacteria and fungi clearly attached. A large number of microorganisms were attached to the cross section, and the small spikes were eroded or had completely disappeared (**Figure 6B**). The outer surface structure of pericarps treated at 37°C was destroyed, and the fibrous villi were completely absent in most treatment groups, while lamellar debris was visible. The inner wall structure of the inner surface was damaged, and many cracks and holes were observed, with no obvious textured protrusions. A large number of holes were apparent in the cross section, with a loose structure and a large number of microorganisms attached (**Figure 6C**).

**Figure 7:**
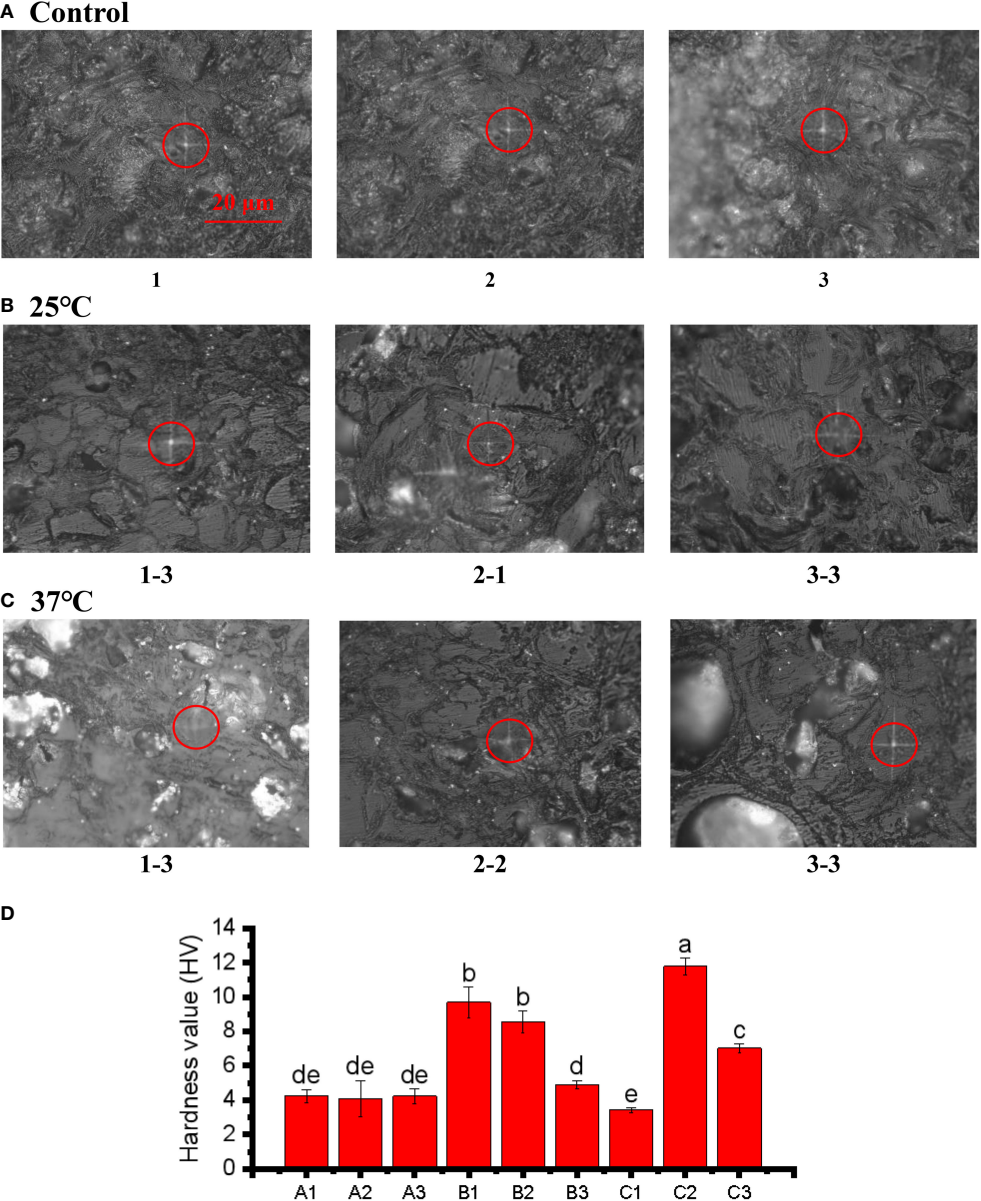
Microindentations (squares in the circles) on the cellular structure of the control **(A)**, 25°C **(B)**, and 37°C treated **(C)** group and three point hardness values **(D)**. The bar of A, B and C was the same size with 20 μm.The small letters represent significant differences (p < 0.05).

The Vickers hardness index was applied to assess the hardness of pericarps. The results revealed that the pericarps in both the 25°C and 37°C treatment groups were harder than those in the control group (**Figure 7A**), although the X-1 sample (1-3) in the 37°C, treatment was slightly less hard. This was consistent with our previous research results. The increasing hardness of the pericarps is an important reason for the cracking of the pericarps (**Figures 7B, C** ). We speculate that the increase in pericarps hardness is one reason for the increase in pericarps brittleness, and the increase in fruit pericarps brittleness leads to cracking of the pericarps. The positions circled in red in the figure are the locations that we drilled to measure the Vickers hardness of pericarps (**Figure 7D**). The light transmissivity on the pericarps in the treatment group also indicated that the texture of the pericarps began to change, and the texture of the pericarps in the treatment group was no longer dense compared with the control group.

**Figure 8:**
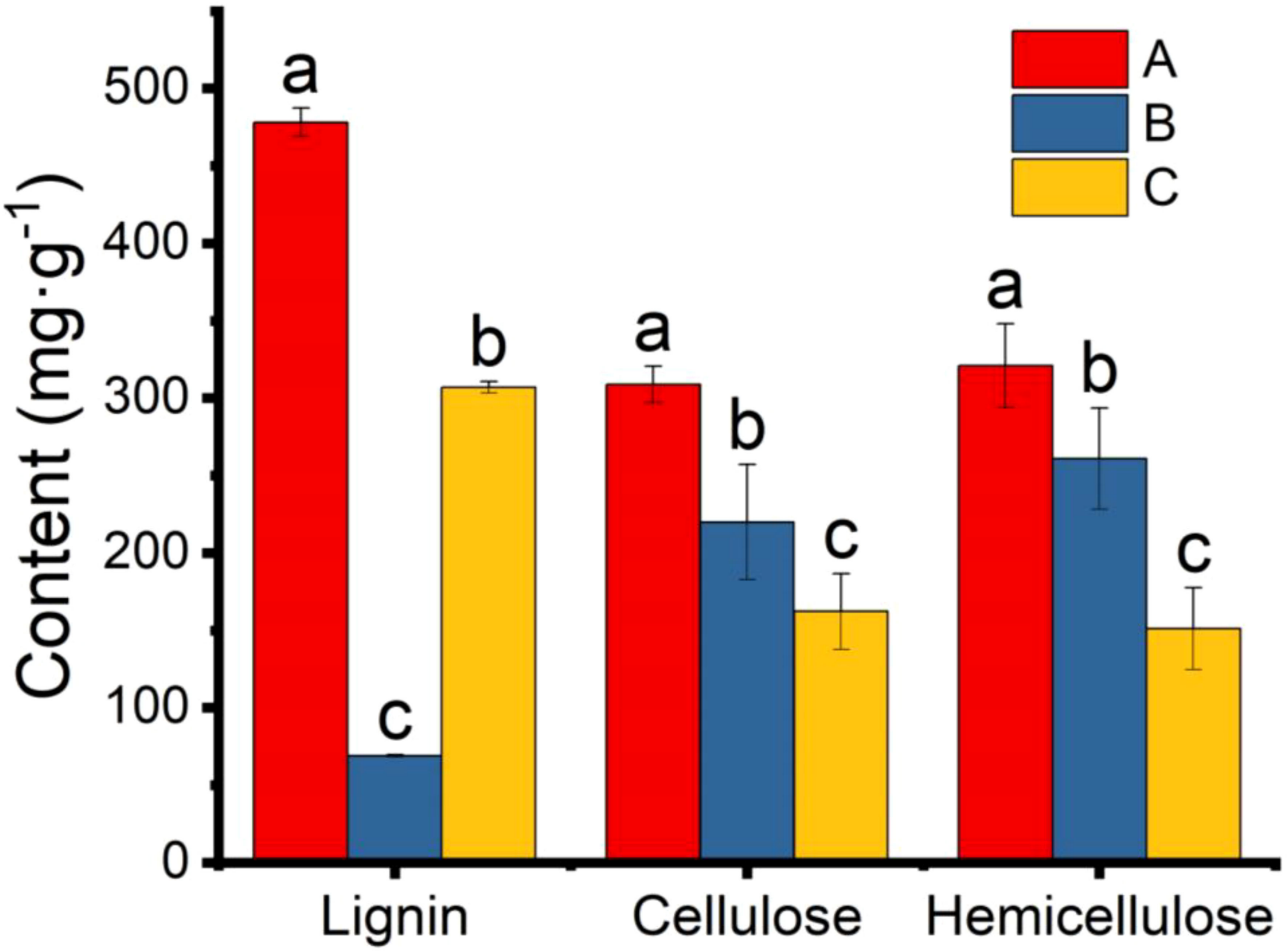
Content of lignin, cellulose, and hemicellulose in pericarp from *Tilia miqueliana* of control (red), 25 °C (blue), and 37 °C (yellow) treatment group. The small letters represent significant differences (p < 0.05).

The main components (cellulose, hemicellulose and lignin) of the pericarp were also determined. The lignin and hemicellulose contents in the 25 °C and 37 °C treatment groups were significantly lower than in the control group (**Figure 8**). The lower lignin and hemicellulose contents indicated that the pericarps were consumed by microbial scarification and the texture of the pericarps became looser. However, the amount of cellulose in both groups increased significantly, contrary to we had expected. We hypothesised that this might be due to an error in the determination of cellulose, which also takes into account the relative composition of the microorganisms attached to the pericarps surface. Assuming that the result was correct and was not a technical error, the high cellulose content may be one of the reasons for the high value of the Vickers hardness index.

### Identification of six target microorganisms

**Figure 9:**
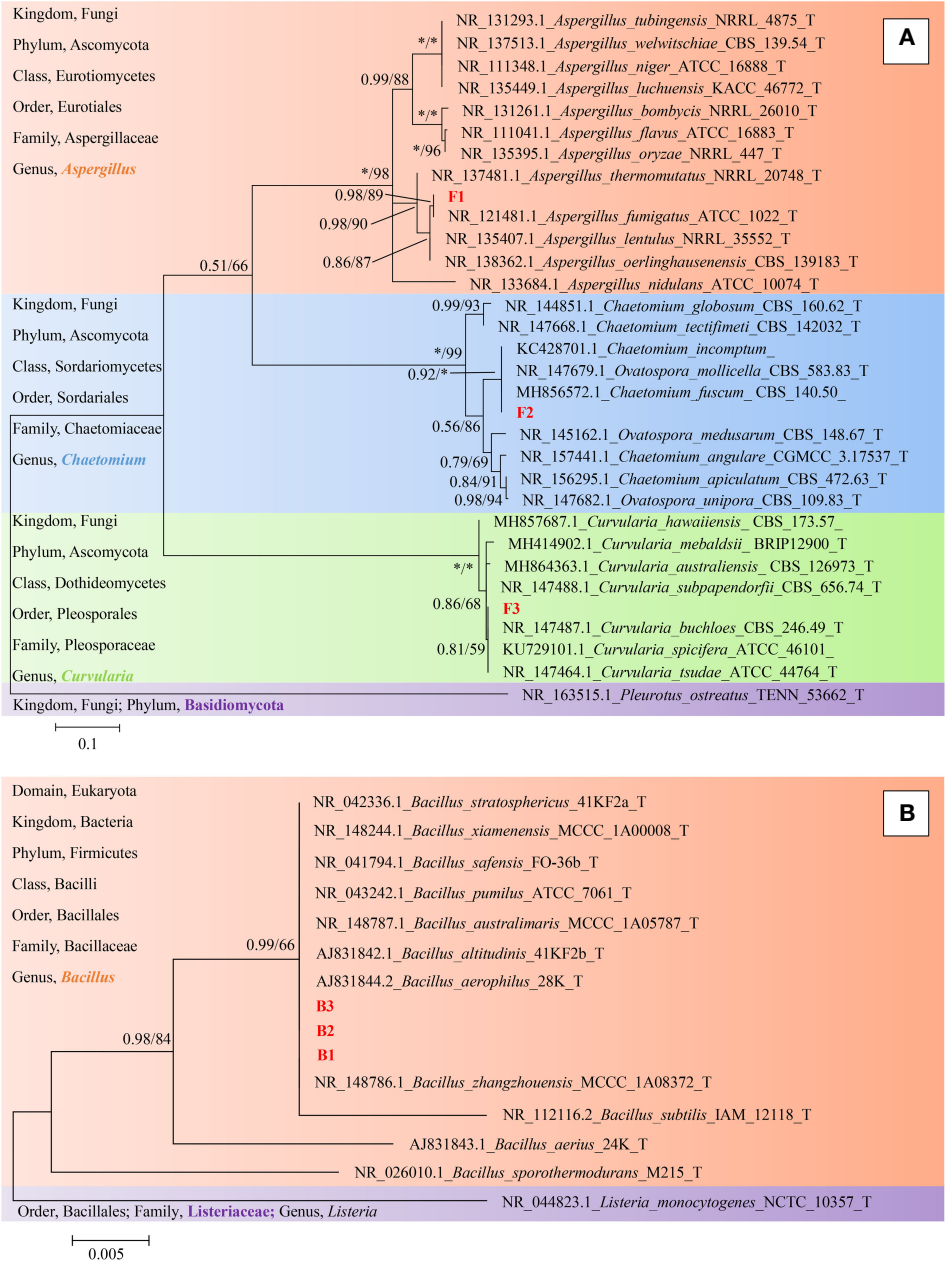
Phylogeny identification of six target microorganisms. On the left side of the figure is a description of the taxonomic status of the species used to construct the phylogenetic tree. **(A)**, Maximum likelihood phylogeny of ITS regions for target fungi classified in Phylum Ascomycota. *Pleurotus ostreatus* (TENN_53662_T) from Phylum Basidiomycota was chosen as out-group. Best-fit model of Bayesian Inference phylogeny according to BIC: SYM + G4; Best-fit model of Maximum likelihood phylogeny according to AIC: Kimura 2-parameter + G + I; alignment, ITS 471 bp. Scale bar: 0.1 substitutions per nucleotide position. **(B)**, Maximum likelihood phylogeny of 16S ribosomal RNA sequence for target bacteria classified in Family Bacillaceae. *Listeria monocytogenes* (NCTC_10357_T) from Family Listeriaceae was chosen as out-group. Best-fit model of Bayesian Inference phylogeny according to BIC: K80 (K2P) + I; Best-fit model of Maximum likelihood phylogeny according to AIC: Kimura 2-parameter; alignment, 16S rRNA 511 bp. Scale bar: 0.005 substitutions per nucleotide position. Support in nodes is indicated above branches and is represented by posterior probabilities (BI analysis) and bootstrap values (ML analysis). Full support (1.00/100 %) is indicated with an asterisk (*). Bootstrap values lower than 50 is hidden. T indicates ex type. The strain F1, F2, F3 and B1, B2, B3 with red font were the strains to be identified.

**Figure 10:**
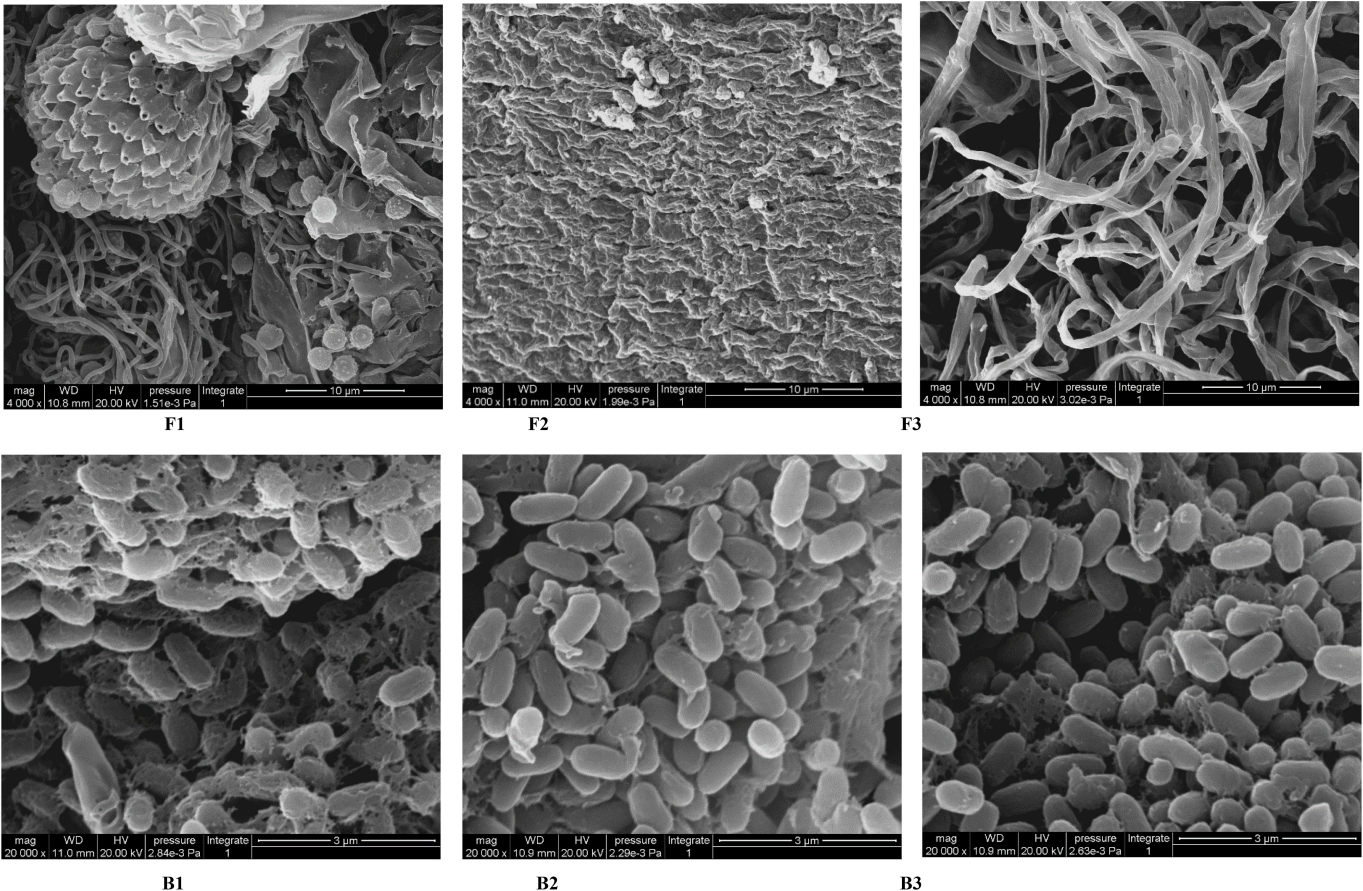
SEM images of target microorganisms. Fungi F1, F2, F3 and Bacteria B1, B2, B3. The bar size is 10 μm for F1-F3 and 3 μm for B1-B3.

Finally, representative fungi (three isolates) and bacteria (three isolates) were randomly selected for screening and identification. *Listeria monocytogenes* (NCTC_10357_T) from the order Bacillales, family Listeriaceae and genus *Listeria* was selected as the out-group of bacteria species from genus *Bacillus* and *Pleurotus ostreatus* (TENN_53662_T) from phylum Basidiomycota was selected as the out-group of fungi species from phylum Ascomycota. **Figures 9A, B** presented the respective phylogenetic relationships of three bacteria species and three fungi species in maximum likelihood (ML) trees, which were constructed using ITS region and 16S ribosomal RNA sequence markers (**Figure 9**). The 16S sequence of three bacteria species were clustered with corresponding sequences from other type strains of genus *Bacillus* (BI= 0.99, bootstrap = 66%). The ITS region sequences of three fungi species were clustered with the corresponding sequences from other type strains of genera *Aspergillus* (BI= 0.98, bootstrap = 89%), *Chaetomium* (BI= 0.92, bootstrap =100%) and *Curvularia* (BI= 0.81, bootstrap = 59%), respectively. A phylogenetic tree of the six strains revealed that all three bacteria species were included in the genus *Bacillus*, and the three fungi species were from three genera *Aspergillus*, *Chaetomium* and *Curvularia*. Through scanning electron microscope (SEM), mycelium and spores, typical organs of fungi in F1 and F3 (**Figure 10**), were observed. Typical morphology of *bacillus* species was observed in bacteria B1, B2 and B3 (**Figure 10**). However, no characteristic morphological organs of fungi were observed in the microscopic images of F2. Since it only had weak mycelia on PDA medium, it was speculated that it was difficult to cultivate or its growth was affected by other unknown factors.

## Discussion

Trees are the main force in the forest ecosystem and are considered the lungs of the earth, so sufficient numbers of trees within forests and stable generations are necessary to maintain a stable climate. However, many species including *T. miqueliana* have a deep dormancy due to their hard and impermeable seed coats or pericarp (e.g. *Sapindus mukorossii*, *Sinojackia xylocarpa*), which increases the length of the life cycle and makes breeding difficult (Shi et al., 1999; Shi, 2006; Thapa and Gautam, 2006). Physical and chemical methods have been applied to break the dormancy of hard coat seeds. Physical methods mainly involve the manual removal of the hard pericarp, while chemical methods may involve applying sulfuric acid or an alkaloid to open the permeability or mechanical barrier of the seed coat or pericarp (Barton, 1965; Yang and Yin, 2006). The existing methods used to eliminate the hard seed coat or pericarp are time-consuming and expensive, and are not suitable for large-scale breeding.

In the absence of human intervention, seeds in nature will normally remain dormant for a long period of time after they fall to the soil surface (Chambers and MacMahon, 1994). When a seed undergoes morphological or physiological post-ripening, the dormancy of the seed can be broken and the seed can germinate when external conditions are suitable (Bewley, 1997). Similarly, artificial processes, such as stratification, can enable morphological and physiological maturation while ensuring that seeds are sterile. It is very common for seeds of different species that fall into the soil to decay to different degrees, and seeds of the same species can be present in the soil with different degrees of decay. Seeds may rot in soil because their self-protection mechanisms are weak while they are still dormant and they may be spoiled by microorganisms from the outside world. Water is the source of life, and high temperature and humidity are the best conditions for the breeding and reproduction of microorganisms, which will increase the occurrence of microbial scarification. Seeds that can germinate rapidly can activate their own defence and protection mechanism after germination, reducing the chance of being invaded by saprophytic microorganisms (Evans and Etherington, 1990).

Soil is one of the most important factors in an ecosystem and provides a habitat for many different microorganisms. Soil is a huge microbial resource bank, among which saprophytic microorganisms have high diversity in terms of species and functions (Wardle, 2006). Common soil microorganisms, including bacteria and fungi, are important in the decomposition of plant litter (woody and herbaceous) (Aislabie et al., 2013). For the microorganism itself, the decomposition event is a critical part of its feeding behaviour, while the secretion of secondary metabolites can lead to the additional breakdown of substances in the environment (Allison, 1973). Different microorganisms have different preferences for and abilities to degrade plant fibre components (Janusz et al., 2017). The interrelations between different organisms and the results of their interactions have long been a topic of interest. Based on the accumulation of such knowledge from previous research, we considered the feasibility of the application of microbial activity on plant seeds. In screened and controlled conditions, microorganisms can be considered a “good helper.” Some microorganisms in the soil, including bacteria and fungi, can be cultured and easily used. We attempted to screen out some microorganisms that could rapidly break down the pericarps of hard-pericarped seeds (e.g. *T. miqueliana*, *S. xylocarpa*). The decomposition of seeds and pericarps by microbes can lead to both positive and negative results, and how microbes are viewed and treated determines their role and identity relative to their beneficiaries and victims (Sperber et al., 2017). The key is to know whether the microbes used to erode pericarps will indiscriminately damage the embryo, including the germ and endosperm within it. Therefore, determining the correct microorganisms and controlling the degree of microbial erosion of the pericarp is a key issue.The pericarp is a physical barrier and mechanical constraint to seed germination. The permeability of the pericarp and seed coat influences communication between the seed and the outside world and controls water entry. Mechanical restraint of the pericarp and seed coat hinders the enlargement of the volume and germination of the seed after absorbing water (Steinbrecher and Leubner-Metzger, 2018). Cracking of the fruit pericarps enables seed germination, and water content, air humidity and oxygen flow all affect the cracking of pericarps in soil. Considering the diversity and individual specificity of seeds, it is necessary to collect microorganisms from different isolated sources and living conditions. In this study, we used four soil sample treatments to screen for various kinds of microorganisms that could erode the fruits. According to their function and adaptability, the microorganisms screened under different treatment methods were expected to fall into the following categories: microorganisms suitable for drought conditions; microorganisms suitable for moist conditions; microorganisms that can decompose the pericarps of seeds, such as saprophytic fungi (which eat dead pericarps), white rot fungi (which eat lignin) and brown rot fungi (which eat cellulose); and microorganisms that can promote seed germination.

Some recent seed germination studies have shown that using sulfuric acid to corrode the pericarps can improve the permeability of the pericarp and seed coat and thereby contribute to seed germination (Mira et al., 2015). Exogenous gibberellin and other hormones can also promote seed germination. We applied various methods to a hard pericarped seed treated by microorganisms to render it suitable for subsequent seed cultivation. To prevent damage to the seeds, the seed coats under the pericarps were removed and then planted. Instead of removing the pericarps that remained after microbial scarification, the entire fruits were planted with the addition of a small amount of fungicide. In the best case, the microbes that were found to eat into the pericarp also had functions that promoted seed germination (such as secreting germination-promoting hormones). As a result, the fruits can be directly planted with microorganisms, eliminating the need for manual processing. Therefore, these microorganisms also have the potential to be developed into a biological fertiliser. Of the three methods mentioned above, the combination of the conventional addition of exogenous hormones before seeding may promote earlier seed germination and reduce the chance of infection. Our findings may represent a breakthrough in the removal of the physical and mechanical barriers that are critical to the germination of hard coat seeds. We expect that the application of this method will have positive effects on the germination of other hard coat seeds. More research will be required to explore the effects on other seeds.

In this manuscript, metagenomics was used to detect the diversity and function of soil microbiome. A new idea and method for detecting artificial mixed microbiome by metagenomic sequencing is proposed for the first time. Here we refer to this approach as metagenomics identification. This experimental concept can even be derived from other aspects. In addition to quickly determining the taxonomic status of a large number of target microbes, this method can also be used to understand other potential functions of target microbes, and it can also be used to obtain functional information of microbes screened for other purposes in other experimental project.

## Conclusions

In this research, the selected microorganisms to degrade the hard pericarp of the fruits without damaging the key structure of the seed were use. Four soil sample treatments were used to screen for various kinds of microorganisms that could erode the fruits. The decomposition process of *T. miqueliana* pericarps in soil was simulated artificially. With reference to the principle of the classical replica plating method, more than 100 different culturable microorganisms that could rapidly erode the pericarp were collected from the surface of pericarps under different culture conditions. In addition, representative 3 fungi isolates and 3 bacteria isolates were randomly selected for screening and identification. At the same time, we successfully extended the concept of metagenomics and applied it to the identification of mixed artificial cultures. Embryos inside the eroded fruits had good viability, and the physical and chemical data also partially revealed the mechanism of pericarp erosion and cracking. Our findings may represent a breakthrough in the removal of the physical and mechanical barriers that are critical to the germination of hard coat seeds. We expect that the application of this method will have positive effects on the germination of other hard coat seeds.

## Data availability statement

The datasets presented in this study can be found in online repositories. The names of the repository/repositories and accession number(s) can be found in the article/supplementary material.

## Author contributions

YS, XW, YW, and XS conceived the idea and led the study design. YW and XS carried out the experiments, performed the analysis, and wrote the manuscript. YW, XS, HP, and CP reviewed and edited the manuscript. All authors contributed to the article and approved the submitted version.
